# Cingulate transcranial direct current stimulation in adults with HIV

**DOI:** 10.1371/journal.pone.0269491

**Published:** 2022-06-03

**Authors:** Xiong Jiang, Sophia Dahmani, Margarita Bronshteyn, Fan Nils Yang, John Paul Ryan, R. Craig Gallagher, Srikanth R. Damera, Princy N. Kumar, David J. Moore, Ronald J. Ellis, Peter E. Turkeltaub

**Affiliations:** 1 Department of Neuroscience, Georgetown University Medical Center, Washington, DC, United States of America; 2 Department of Medicine, Georgetown University Medical Center, Washington, DC, United States of America; 3 Department of Psychiatry, University of California, San Diego, CA, United States of America; 4 Department of Neurosciences, University of California, San Diego, CA, United States of America; 5 Department of Neurology and Center for Brain Plasticity and Recovery, Georgetown University Medical Center, Washington, DC, United States of America; BG-Universitatsklinikum Bergmannsheil, Ruhr-Universitat Bochum, GERMANY

## Abstract

**Background:**

Neuronal dysfunction plays an important role in the high prevalence of HIV-associated neurocognitive disorders (HAND) in people with HIV (PWH). Transcranial direct current stimulation (tDCS)—with its capability to improve neuronal function—may have the potential to serve as an alternative therapeutic approach for HAND. Brain imaging and neurobehavioral studies provide converging evidence that injury to the anterior cingulate cortex (ACC) is highly prevalent and contributes to HAND in PWH, suggesting that ACC may serve as a potential neuromodulation target for HAND. Here we conducted a randomized, double-blind, placebo-controlled, partial crossover pilot study to test the safety, tolerability, and potential efficacy of anodal tDCS over cingulate cortex in adults with HIV, with a focus on the dorsal ACC (dACC).

**Methods:**

Eleven PWH (47–69 years old, 2 females, 100% African Americans, disease duration 16–36 years) participated in the study, which had two phases, Phase 1 and Phase 2. During Phase 1, participants were randomized to receive ten sessions of sham (n = 4) or cingulate tDCS (n = 7) over the course of 2–3 weeks. Treatment assignments were unknown to the participants and the technicians. Neuropsychology and MRI data were collected from four additional study visits to assess treatment effects, including one baseline visit (BL, prior to treatment) and three follow-up visits (FU1, FU2, and FU3, approximately 1 week, 3 weeks, and 3 months after treatment, respectively). Treatment assignment was unblinded after FU3. Participants in the sham group repeated the study with open-label cingulate tDCS during Phase 2. Statistical analysis was limited to data from Phase 1.

**Results:**

Compared to sham tDCS, cingulate tDCS led to a decrease in Perseverative Errors in Wisconsin Card Sorting Test (WCST), but not Non-Perseverative Errors, as well as a decrease in the ratio score of Trail Making Test—Part B (TMT-B) to TMT—Part A (TMT-A). Seed-to-voxel analysis with resting state functional MRI data revealed an increase in functional connectivity between the bilateral dACC and a cluster in the right dorsal striatum after cingulate tDCS. There were no differences in self-reported discomfort ratings between sham and cingulate tDCS.

**Conclusions:**

Cingulate tDCS is safe and well-tolerated in PWH, and may have the potential to improve cognitive performance and brain function. A future study with a larger sample is warranted.

## Introduction

Despite largely successful peripheral viral suppression with combination antiretroviral treatment (cART), HIV-associated neurocognitive disorders (HAND) remain highly prevalent in people with HIV (PWH). In the cART era, neuronal dysfunction and synaptodendritic damage emerge as key mediators of cognitive decline/impairment in PWH [1–3]. Therefore, in addition to targeting neuroinflammation with enhancing cART, alternative approaches targeting neuronal/synaptodendritic function may have the potential to serve as therapeutic tools for HAND intervention and treatment.

Transcranial direct current stimulation (tDCS), a technique that safely and non-invasively modulates brain activity through a subthreshold shift of resting membrane potentials [4], has been shown to improve neuronal and synaptodendritic function [5, 6], suggesting that tDCS may have the potential to serve as an alternative approach for HAND intervention and treatment in PWH. In tDCS, the modulation effects are proposed to depend on polarity, i.e., an increase in excitability with anodal (positive) stimulation versus a decrease in excitability with cathodal (negative) stimulation [7], although this association is likely oversimplified, especially with regards to neurocognitive function [8]. Over the past two decades, there has been exponentially growing interest in applying tDCS to treat various psychiatric and neurological conditions. A comprehensive review concluded that tDCS is probably effective in treating fibromyalgia, depression, and addiction/craving [9]. The efficacy of tDCS in treating depression has been confirmed in a more recent review [10].

Compared to the large number of tDCS studies on many other neurological disorders, however, few studies have examined the effects of tDCS in HIV brain disease. To the best of our knowledge, there are six published works that investigated the safety and efficacy of tDCS in PWH. One open-label study examined the effects of tDCS in treating depression in PWH [11], and five single-blind studies investigated whether a combination of tDCS and cognitive training could improve cognitive performance in PWH [12–16]. In two studies with PWH participants who were older than 50 and met the Frascati criteria for HAND diagnosis, Ownby and colleagues investigated the effects of anodal tDCS over the left dorsal lateral prefrontal cortex (DLPFC) while participants were receiving a computer-based cognitive training [12, 16]. Subjectively, PWH who received active tDCS tended to rate the intervention trials more positively. However, objective neuropsychological tests did not find any significant difference between active and sham tDCS [12, 16]. In a series of tDCS studies combined with speed of processing training, Cody, Fazeli, Pope, Vance, and colleagues investigated the effects of anodal tDCS over the right DLPFC in older PWH (aged 50 and older) and age-matched HIV-uninfected controls: compared to sham tDCS, active tDCS led to a stronger improvement in psychomotor speed [15], cautious driving behavior [13], and oral reading [14] in both HIV-infected and HIV-uninfected adults. Overall, these findings are encouraging but also suggest a need for additional research, i.e., applying tDCS to different brain regions.

In this study, the anterior cingulate cortex (ACC) and the posterior cingulate cortex (PCC) were selected as the target regions for anodal tDCS neuromodulation, with a focus on the dorsal ACC (dACC). The selection of ACC as the primary target region was motivated by a neural model of HAND we recently proposed [17], which recognizes ACC as one of the most commonly affected brain regions in PWH and proposes an important role of ACC injury in HAND progression—suggesting that the ACC may serve as a potential therapeutic target for HAND intervention and/or treatment. The important role of ACC in HIV brain disease is supported by neurobehavioral and brain imaging studies in HIV: first, ACC injury in PWH has been detected using various brain imaging techniques, including structural MRI [17], task-based functional MRI (fMRI) [18], task-free fMRI [19] and perfusion MRI [20], proton magnetic resonance spectroscopy (MRS) [3, 21–23], and positron emission tomography (PET) [20, 24]; second, the high prevalence of executive deficits [25] and apathy [26]—both are known to involve the ACC [27–29]—provides additional evidence supporting the prevalence and importance of ACC injury in PWH. Furthermore, using invasive and non-invasive brain stimulation techniques (including deep brain stimulation, repetitive transcranial magnetic stimulation (rTMS), and tDCS), previous studies provided evidence suggesting that dACC stimulation might be effective in modulating brain/cognitive function [30–33] and emotional processes [34], as well as in treating disorders like obsessive compulsive disorder [35, 36]. Highly relevant to the present study, two previous studies found that one session of anodal tDCS stimulation (1.5–2 mA, 15–20 minutes) targeting the dACC was sufficient to induce detectable changes at a behavioral (“more efficient adjustments in decision‐making strategies” [33]) or neural level (neuronal signal related to “improved efficiency of neural resources for inhibitory control and error processing” [32]), providing further support for the present study. The posterior cingulate cortex (PCC) was selected as the secondary target region, as the PCC is a central hub of the default mode network (DMN) and both PCC and DMN have been frequently implicated in various neurodegenerative diseases [37, 38], including HAND [19, 39, 40].

Applying anodal tDCS over the ACC and the PCC, here we conducted a randomized, *double-blind*, partial crossover, and placebo-controlled pilot study to test the hypothesis that the cingulate cortex (especially the dACC) is a potential therapeutic target in adults with HIV. Neuropsychological and MRI data, along with self-reported discomfort ratings, were collected to test three predictions: i) cingulate tDCS is safe and well-tolerated in adults with chronic HIV-disease (primary goal); ii) cingulate tDCS might lead to improvements in cognitive function (secondary goal); and iii) cingulate tDCS might result in a change in brain structure and/or brain function (secondary goal).

## Methods

### Participants

Fifteen PWH from the greater Washington D.C. metropolitan area were enrolled into the study. Eleven of them finished the study prior to the COVID-19 pandemic (age 47–69 years old, 100% African American, 2 females) (Table 1 and S1 Table). One participant with MRI contraindications was excluded from the MRI portion of the study, but participated in all other aspects of the study (S1 Table). Written informed consent approved by the Institutional Review Board at Georgetown University Medical Center was obtained prior to enrollment. Medical data and other comorbidities such as substance abuse were assessed. Viral load and current CD4 counts were collected using their most recent medical records. CD4 nadir and estimated duration of HIV disease were collected through self-report.

**Table 1:** The demographics and study assignment of the participants.

	tDCS (n = 7)	Sham (n = 4)	*p*
**Age at Baseline, years**	57 (4)	59.5 (5.5)	*n*.*s*.
**Gender, % Male**	85.7	75.0	*n*.*s*.
**Education, years**	13 (3)	14 (1)	*n*.*s*.
**Disease Duration, years**	30 (3.5)	29 (4.25)	*n*.*s*.
**On stable cART (%)**	100%	100%	*n*.*s*.
**Virally suppressed (%)**	100%	^1^75%	*n*.*s*.
**CD4 at Baseline, cells/uL**	680.5 (296.75)	893 (719.75)	*n*.*s*.
**Nadir CD4, cells/uL**	250 (285.25)	59 (92.25)	*n*.*s*.
**Pain Scale—Phase 1**	0 (0.05)	0 (1)	*n*.*s*.
**Pain Scale—Phase 2** ^**2**^	-	0.2 (0.8)	*-*

During Phase 1, participants were randomized to receive 10 sessions of active cingulate tDCS (n = 7) or sham tDCS (n = 4). The treatment assignments were unknown to the participants and the technicians who administered tDCS. During Phase 2, the four participants in the sham tDCS group received open-label active cingulate tDCS. Data are presented as median (IQR) or %.

^1^ Virally suppressed is defined as a plasma viral load less than 50 copies/mL and one participant in the Sham group had a plasma viral load of 6353 copies/mL.

^2^ During Phase 2, only active cingulate tDCS was administered to participants.

### Study design (randomized, double-blind, placebo-controlled, partial crossover)

**Fig 1:**
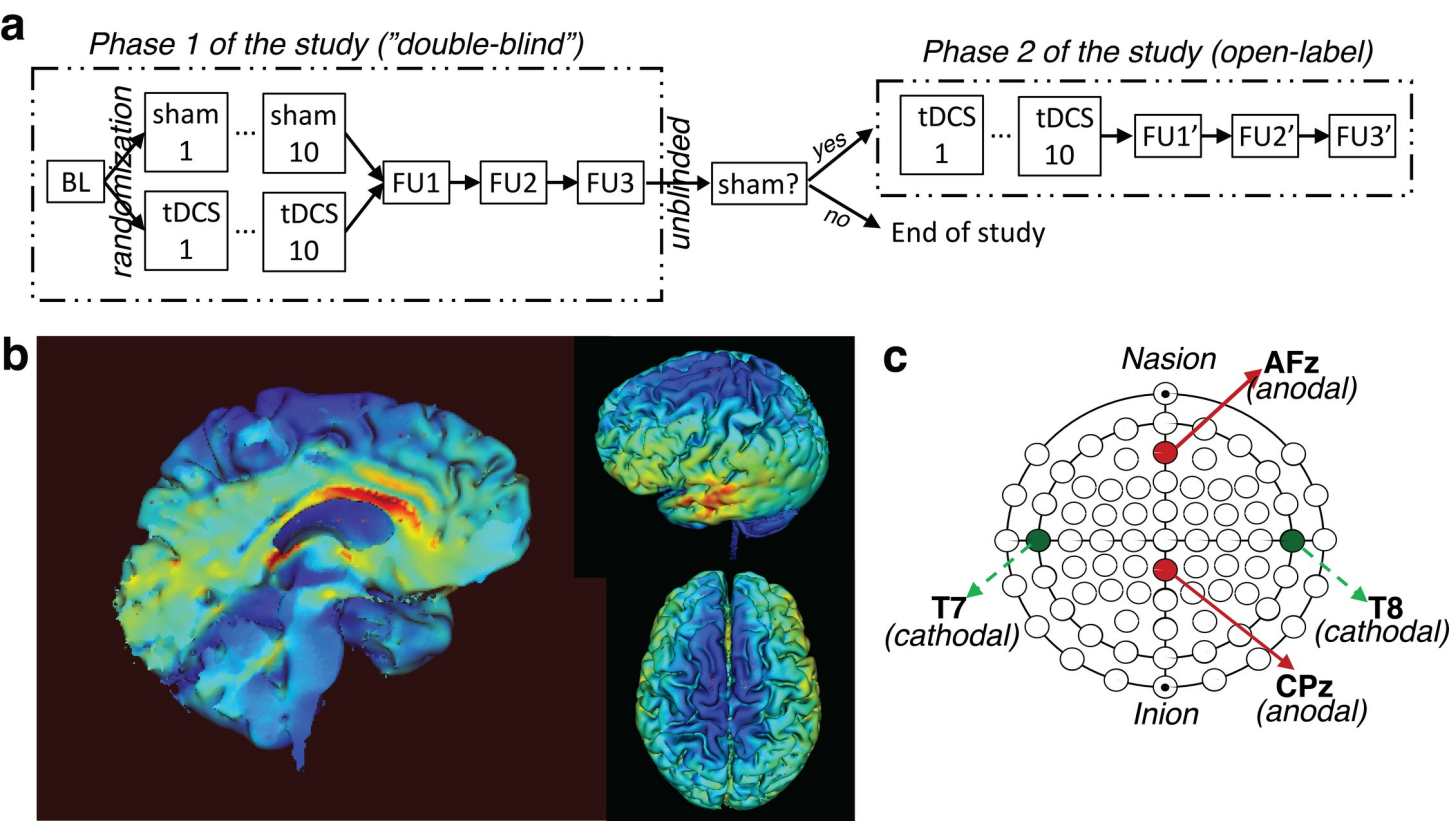
Experimental design and tDCS simulation and setup. **(a)** This partial crossover study had two study phases. During Phase 1, participants were randomly assigned to receive sham or cingulate tDCS based on age and sex at birth. The treatment assignments were unknown to the participants as well as the study team during Phase 1. There were ten tDCS treatment sessions, with one per weekday over the course of 2–3 weeks. Neuropsychology and MRI data were collected from four additional study visits, including one baseline visit prior to the first tDCS treatment session (BL) and three follow-up visits (FU1/FU2/FU3, which were approximately one-week, three-weeks, and three-months after the last tDCS treatment session, respectively). After the FU3 visit, the treatment assignments were unblinded. The study was finished for participants who received active cingulate tDCS during Phase 1. In contrast, participants who received sham tDCS during Phase 1 were invited back to participate in Phase 2 of the study (open-label cingulate tDCS treatment). The study design of Phase 2 was identical to that of Phase 1, except that FU3 from Phase 1 served as BL for Phase 2. **(b)** Model of the induced electrical field on a typical brain, shown as field intensity (V/m) irrespective of the direction of current flow (the range of field intensity: 0 to 0.43 V/m). **(c)** The locations of anodal and cathodal electrodes with a 2x2 high-definition tDCS setup, which were based on the simulations in (**b**). Electrode locations were based on the international 10–10 electroencephalography (EEG) system.

There were two study phases: Phase 1, and Phase 2 (Fig 1A). During Phase 1, participants were randomized to receive active cingulate tDCS or sham tDCS (see Cingulate tDCS section below). Both involved 10 treatment sessions (one session per weekday) over the course of two to three weeks (11.7±1.7 weekdays). The treatment assignments were unknown to the participants and the technicians who administered tDCS (“*double-blind*”). Neuropsychological and MRI data were collected at four additional study visits to evaluate treatment effects, including one baseline visit (BL, prior to the first tDCS treatment session), and three follow-up visits (FU1/FU2/FU3, corresponding to ~1-week, ~3-week, and ~3-month after the last tDCS session, respectively).

After the FU3 study visit, the treatment assignment of Phase 1 was unblinded. The study was finished for participants who received active cingulate tDCS, whereas participants who received sham tDCS were invited back to participate in Phase 2 of the study, which involved open label cingulate tDCS. The experimental design of Phase 2 was identical to that of Phase 1, except the FU3 study from Phase 1 served as the BL study visit for Phase 2 (Fig 1A).

### Cingulate tDCS

Cingulate tDCS and sham tDCS were applied using a custom designed Soterix HD-tDCS- CT unit stimulator (Soterix Medical, Inc.) when participants were at rest. The stimulator consists of a current source powered by 9-Volt batteries, pre-programmed with >200 random treatment codes that can be used for studies with a double-blind stimulation design, such as Phase 1 of this study. Ag/AgCl sintered ring electrodes were used with electroconductive gel to make contact with the scalp. To target the ACC and the PCC regions, Soterix Medical Individualized Modeling Service (https://soterixmedical.com/research/software/modeling-service) was requested to develop an electrode montage to provide anodal stimulation to dACC and PCC, while minimizing possible inhibitory (cathodal) stimulation of potentially important structures in the fronto-parietal brain networks. Electrode locations are based on the international 10–10 electroencephalography (EEG) system. Based on finite element electrical field models (Fig 1B), the two anodal electrodes were placed at AFz and CPz, with two return (cathodal) electrodes at T7 and T8, respectively (Fig 1C). One participant reported a burning sensation at the CPz location after the first active tDCS session. The electrode was moved from CPz to Pz for the rest of nine active tDCS sessions and the participant reported no burning sensation after the switch.

In cingulate tDCS conditions, the current was ramped up to 1.5mA over 30sec, applied for 20 minutes, and then ramped down over 30sec [41, 42]. The dosage was based on the recommendations from an expert panel [43] and the findings from a comprehensive safety review study [44]. The sham tDCS stimulation consisted of an initial 30-second ramp-up to 1.5 mA, followed immediately by a 30-second ramp-down. No current was delivered until 19 minutes later, when another 30-second ramp-up began, followed by a 30-second ramp-down. The ramp up and down after 20 minutes simulates the change in sensation participants sometimes report during active tDCS as the current is ramped down at the end of treatment. All other procedures were identical between the sham and cingulate tDCS conditions. Usually, participants quickly acclimated to the tingling sensations of active tDCS, so sham tDCS was designed to generate these initial tingling sensations, then stop stimulation before delivery of clinically meaningful stimulation. After each sham or cingulate tDCS session, participants reported their discomfort ratings using the Wong-Baker FACES Pain Rating Scale (https://wongbakerfaces.org), then participated in an approximately 20-minute cognitive training session with various designs of cognitive control tasks that are known to engage the dACC region and other executive regions/networks, including go/no-go and task switch [18, 45]. For the purpose of generalizing training effects and stimulating the dACC and associated brain networks/regions, a total of five tasks with different experimental designs were implemented for cognitive training, including two go/no-go tasks (one with letters as the target/distractor; the other with colorful bars as the target/distractor), two tasks with switching-rules (one with line orientation task; the other with animal size task), and one verbal fluency task with alternating rules (i.e., alternating between semantic verbal fluency and letter verbal fluency). However, after each tDCS session, only one of the five tasks was administered. Therefore, with a total of five different tasks and a total of 10 tDCS sessions per study phase, each task was repeated twice during each study phase.

### Neuropsychological testing

During the baseline study visit (BL) and the three follow-up study visits (FU1/FU2/FU3) within each study phase, participants underwent a comprehensive neuropsychological assessment comprised of 12 standardized tests that assessed seven neurocognitive domains that are often affected in PWH, including speed of information processing, verbal fluency, learning, delayed recall, executive function, working memory, and motor abilities. This neuropsychology battery was adopted from the CHARTER study [25] and has been used in our previous studies with larger sample sizes [46, 47]. Additionally, participants completed a modified version of the Lawton and Brody Activities of Daily Living questionnaire (1969) in which they self-reported any declines on everyday tasks (e.g., managing finances, managing medications, etc.). The comprehensive neuropsychology battery was administered to all but one participant (S1 and S2 Tables).

Based on the tDCS simulation data (Fig 1B) as well as the cognitive training after each tDCS session (see Cingulate tDCS section above), we limited statistical analyses to four test scores from three neuropsychology tests that have been widely used to assess executive function in PWH, including Wisconsin Card Sorting Test (WCST-64) (Perseverative Errors, and Non-Perseverative Errors), Stroop Color and Word Test (SCWT) (color-word score), and the ratio of Trail Making Test—Part B (TMT-B) to Trail Making Test—Part A (TMT-A) score. WCST is the most commonly used neuropsychological test to examine and assess frontal (including ACC) dysfunction. Compared to other common WCST measures such as Non-Perseverative Errors (or random errors), Perseverative Errors, in which participants continue to use the same old and incorrect strategy even after receiving negative feedback from the previous trial, are probably the best predictor of frontal/ACC dysfunction [48]. The SCWT is another widely used neuropsychological test to assess executive function, and the color-word score—measured as the number of correctly named ink-colors under the incongruent condition (i.e., the word “red” is printed in green)—is used to assess the ability to inhibit cognitive interference [49]. Compared to Trail Making Test–Part A (TMT-A), a set-switching component is added to the TMT-B to assess executive control and cognitive flexibility [50]. The ratio of time, TMT-B / TMT-A, was used in data analysis [51].

### MRI data acquisition

Structural MRI and resting-state functional MRI (fMRI) were acquired at the local institute using a 3-Tesla Siemens Magnetom Trio with a 12-channel head coil or Prisma-Fit scanner with a 20-channel head coil with the same scanning parameters. Structural images were acquired with a 3D T1-weighted sequence (MPRAGE, magnetization prepared rapid acquisition gradient echo) with the following parameters: TR/TE = 1900/2.52ms, TI = 900ms, flip angle = 9°, 160 contiguous 1mm sagittal slices, FoV = 256x160x256mm, 1mm^3^ resolution. One run of resting state fMRI images was acquired with an echo-planar sequence with the following parameters: flip angle = 90°, TR/TE = 2040/29ms, FoV = 205mm (64×64 matrix), 35 interleaved axial slices (4mm thick, no gap; 3.2×3.2mm^2^ in plane resolution). There were 264 acquisitions, and the first 5 acquisitions were discarded prior to preprocessing. In previous studies [46, 47], we found that the scanner upgrade did not have a strong impact on structural and resting state fMRI data, and whether or not including the scanner type as a covariate did not change the conclusions of these studies. In addition, MRI data analyses were limited to participants who received active cingulate tDCS during Phase 1, and for these participants, the same scanner and head coil was used to acquire data at all four study visits (except two participants, s2 and s3, with whom the same scanner was used at BL, FU1, and FU2, but not at FU3). Therefore, the scanner status was not included as a covariate in data analyses, although we did conduct post-hoc analyses with scanner being included as a covariate, which produced similar results that led to the same conclusion.

### MRI data preprocessing and analysis

MRI data preprocessing and analysis was performed using the SPM12 software package (https://www.fil.ion.ucl.ac.uk/spm/software/spm12/) and the CAT12 toolbox release 12.6 (http://dbm.neuro.uni-jena.de/cat/) in MATLAB (release 2018b, The MathWorks, Inc., Natick, Massachusetts, United States). Standard preprocessing procedures were used, including correction for bias-field inhomogeneities, denoising, skull-stripping, segmentation, and corrections for partial volume estimation. Segmentation output comprised GM, WM, and CSF tissue class volumes for each participant. Tissue class volumes were then used to spatially normalize all images to the template in standard Montreal Neurologic Institute (MNI) space. For GM volume-based analyses, normalized GM voxel values were modulated to preserve voxel-wise estimates of the absolute amount of tissue, then smoothed using a Gaussian kernel of 8mm FWHM prior to statistical analysis.

For resting-state fMRI, raw images were first preprocessed in SPM12. The preprocessing consisted of slice-timing correction, realignment, coregistration to structural volume, normalization based on structural normalization parameters obtained from CAT12, outlier identification, smoothing with an 8-mm FWHM. Then, normalized images were processed following the standard CONN pipeline [52], including movement regression, removal of signals from CSF and white matter, band passing [0.009 0.08] Hz, detrend, and a structural aCompCor strategy.

In addition, the software package FreeSurfer v6.0 (https://surfer.nmr.mgh.harvard.edu/) was used to extract cortical thickness estimates using the default longitudinal pipeline [53]. Briefly, an unbiased within-subject template space and image was created using robust, inverse consistent registration. Several processing steps, such as skull stripping, Talairach transforms, atlas registration as well as spherical surface maps and parcellations were then initialized with common information from the within-subject template, significantly increasing reliability and statistical power [53]. Thickness data were then smoothed using a 12mm FWHM kernel prior to statistical analysis.

### Statistical analyses

Three comparisons were conducted independently to investigate treatment effects of active cingulate tDCS, including BL vs FU1, BL vs FU2, and BL vs FU3, respectively.

Neurobehavioral data were analyzed using a robust non-parametric equivalent of repeated-measures ANOVA for mixed factorial designs [54] as implemented in the R package “nparLD” [55]. We assessed the impact of tDCS treatment assignment on test scores across study visits for BL vs FU1, BL vs FU2, and BL vs FU3. For each of these 2x2 factorial models, ANOVA-type statistics were generated to test the null hypothesis of no whole-plot treatment effect, no time effect, and no interaction between these factors. Only the data from Phase 1 (“double-blind”) were analyzed (though the treatment assignments were already unblinded prior to data analysis). The data from Phase 2 were presented for illustration purposes only. Based on the tDCS simulation data (Fig 1B) as well as the cognitive training after each tDCS session (see above), we limited statistical analyses to four test scores from three neuropsychology tests that are designed to examine executive function, including Wisconsin Card Sorting Test (WCST) (Perseverative Errors, and Non-Perseverative Errors), Stroop Color and Word Test (SCWT) (color-word score), and the ratio score of TMT-B to TMT-A (ratio of total time). As a pilot study, all *P* values were reported without correction for multiple comparisons.

MRI data analyses were limited to the participants who received cingulate tDCS during Phase 1, as only two participants who received sham tDCS during Phase 1 had resting state fMRI data eligible for data analysis (S1 Table).

Gray matter volume (GMv) and cortical thickness data analyses were conducted within the CAT or FreeSurfer software package, respectively, using a longitudinal design. A voxel/vertex threshold of *p*<0.005 (uncorrected) along with a cluster threshold of p<0.05 (FWE corrected) was used in both GMv and cortical thickness data analyses.

For resting state fMRI data, seed-to-voxel functional connectivity (FC) analyses were performed to investigate the treatment effect using the dorsal ACC (bilateral, brainnetome atlas 179+180) and the PCC (bilateral, AAL) as the seed regions, respectively (thresholded at voxel-wise *p*<0.01 uncorrected, cluster-wise *p*<0.05 FDR corrected). For clusters that survived the threshold, we extracted the FCs between the clusters and the corresponding seed regions for all participants and all sessions.

## Results

### Participants

Seven participants received cingulate tDCS during Phase 1, and four participants received sham tDCS during Phase 1 then cingulate tDCS during Phase 2 of the study (Table 1 and S1 Table). Non-parametric t-tests implemented using the R package “nparcomp” [56] revealed no significant difference in demographics between the two participant groups.

In addition, there was no significant difference in self-reported discomfort ratings between sham and active cingulate tDCS during Phase 1, nor between sham tDCS during Phase 1 and active cingulate tDCS during Phase 2 (Table 1).

### Neuropsychological data

**Fig 2:**
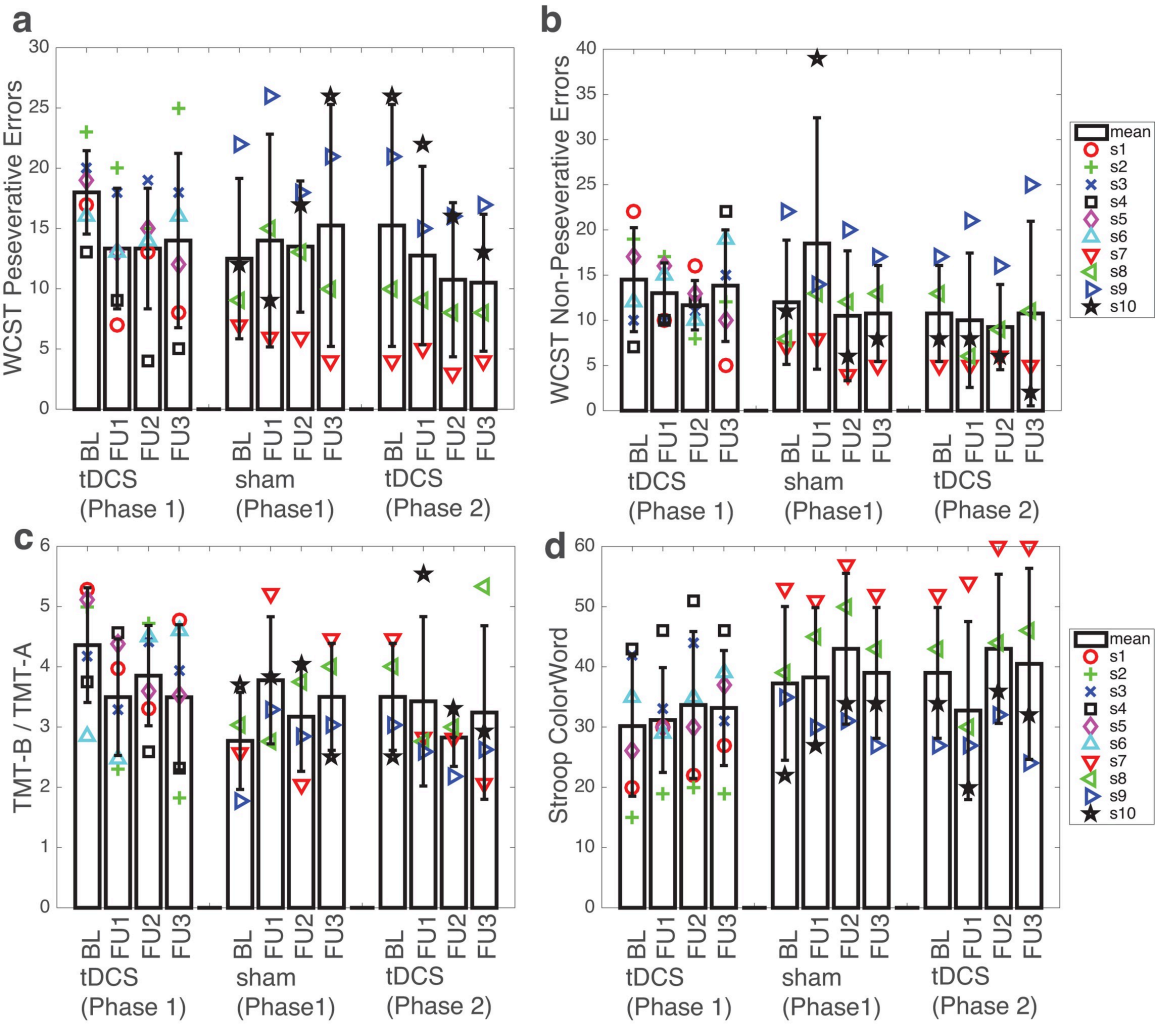
Neuropsychology test scores. **(a)** Wisconsin Card Sorting Test (WCST) Perseverative Errors. **(b)** WCST Non-Perseverative Errors. **(c)** The ratio of Trail Making Test B (TMT-B) to Trail Making Test A (TMT-A). **(d)** Stroop Color and Word Test (SCWT) color-word. Left: tDCS (1st phase)–participants who received cingulate tDCS treatment during Phase 1; middle: sham (1st phase)–participants who received sham tDCS treatment during Phase 1; right: tDCS (2nd phase)–participants who received cingulate tDCS treatment during Phase 2. BL, baseline visit (prior to the first treatment sessions); FU1/FU2/FU3, follow-up visit 1, 2, 3 after the last treatment session, respectively. For participants who received sham tDCS during Phase 1, data from FU3 of Phase 1 were used as BL for Phase 2. Markers represent individual participants. Bars represent group average at each study phase. Error bars represent standard deviation. See Supplementary Materials for the complete test scores of all neuropsychology tests.

For WCST Perseverative Errors (Fig 2A), non-parametric mixed-design ANOVAs revealed a significant interaction between treatment assignments and study visits for BL vs FU1 (F(1,8) = 6.842, p = 0.009) and BL vs FU2 (F(1,8) = 5.745, p = 0.017), but not for BL vs FU3 (F(1,8) = 2.955, p = 0.086). There were no significant main effects of study assignments (at least p>0.107) or study visits (at least p>0.379) for any of the three comparisons (BL vs FU1, BL vs FU2, or BL vs FU3). Post-hoc non-parametric pairwise comparisons revealed that both significant interactions were driven by a decrease in Perseverative Errors after active cingulate tDCS (BL vs FU1, p<0.001; BL vs FU2, p<0.001).

For WCST Non-Perseverative Errors (Fig 2B), there were no significant effects of study assignments (at least p>0.353), study visits (at least p>0.417), or interactions between the two factors (at least p>0.301), for any of the three comparisons (BL vs FU1, BL vs FU2, and BL vs FU3).

For the ratio of TMT-B to TMT-A score (Fig 2C), there was a significant interaction between treatment assignments and study visits for BL vs FU1 (F(1,8) = 5.379, p = 0.020), but not for BL vs FU2 (F(1,8) = 2.606, p = 0.106) or BL vs FU3 (F(1,8) = 3.237, p = 0.072). There was a significant main effect of study assignments for BL vs FU2 (F(1,8) = 6.112, p = 0.013) and BL vs FU3 (F(1,8) = 4.422, p = 0.035), but not for BL vs FU1 (F(1,8) = 1.976, p = 0.160). There were no significant main effects of study visits (at least p>0.841) for any of the three comparisons (BL vs FU1, BL vs FU2, or BL vs FU3).

For SCWT Color-Word score (Fig 2D), there were no significant effects of study assignments (at least p>0.335), study visits (at least p>0.071), or interactions between the two factors (at least p>0.787), for any of the three comparisons (BL vs FU1, BL vs FU2, and BL vs FU3).

**Fig 3:**
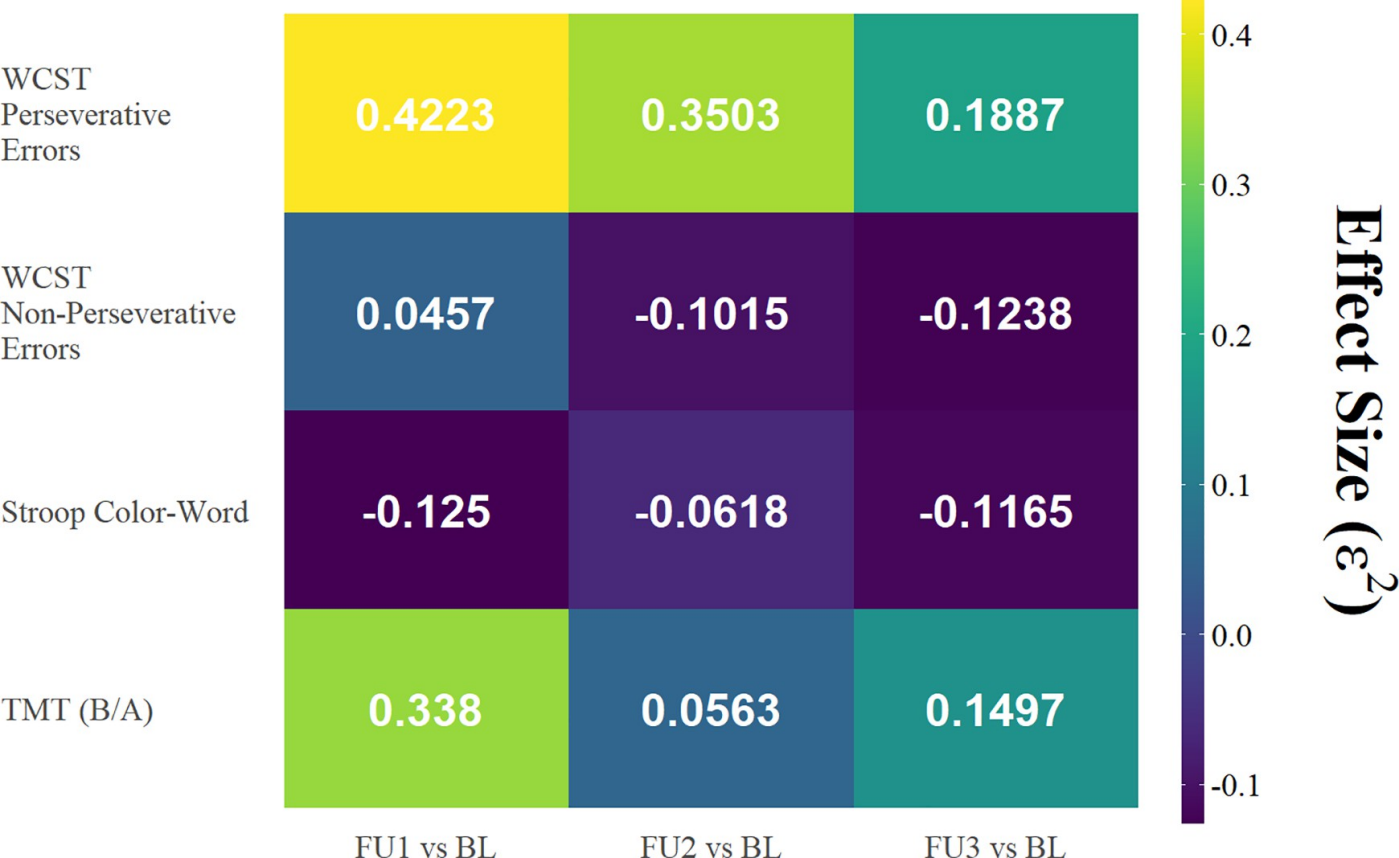
Effect size (epsilon-squared). Separate mixed-design ANOVAs were performed in order to estimate the effect size for the time x condition interaction term at each level of comparison for each neuropsychology test score of interest.

In addition, using the output from the *nparLD* package, we calculated the time (post- vs pre-treatment) x condition (sham vs active tDCS) effect sizes (*epsilon-squared* [57]) for all three comparisons (BL vs FU1, BL vs FU2, and BL vs FU3) and for all neuropsychological test scores listed above (Fig 3, Phase 1 only). The epsilon-squared effect sizes for WCST Perseverative Errors were greater than 0.14 (which denotes a large effect size) at all three comparisons (BL vs FU1, BL vs FU2, and BL vs FU3). Effect sizes for the ratio of TMT-B to TMT-A score were large (ε^2^> = 0.14) at the comparison of BL vs FU1 and BL vs FU3, but not BL vs FU2.

### Structural MRI and resting state Functional Connectivity (FC)

There were no significant changes in gray matter volume or cortical thickness after cingulate tDCS treatment.

**Fig 4:**
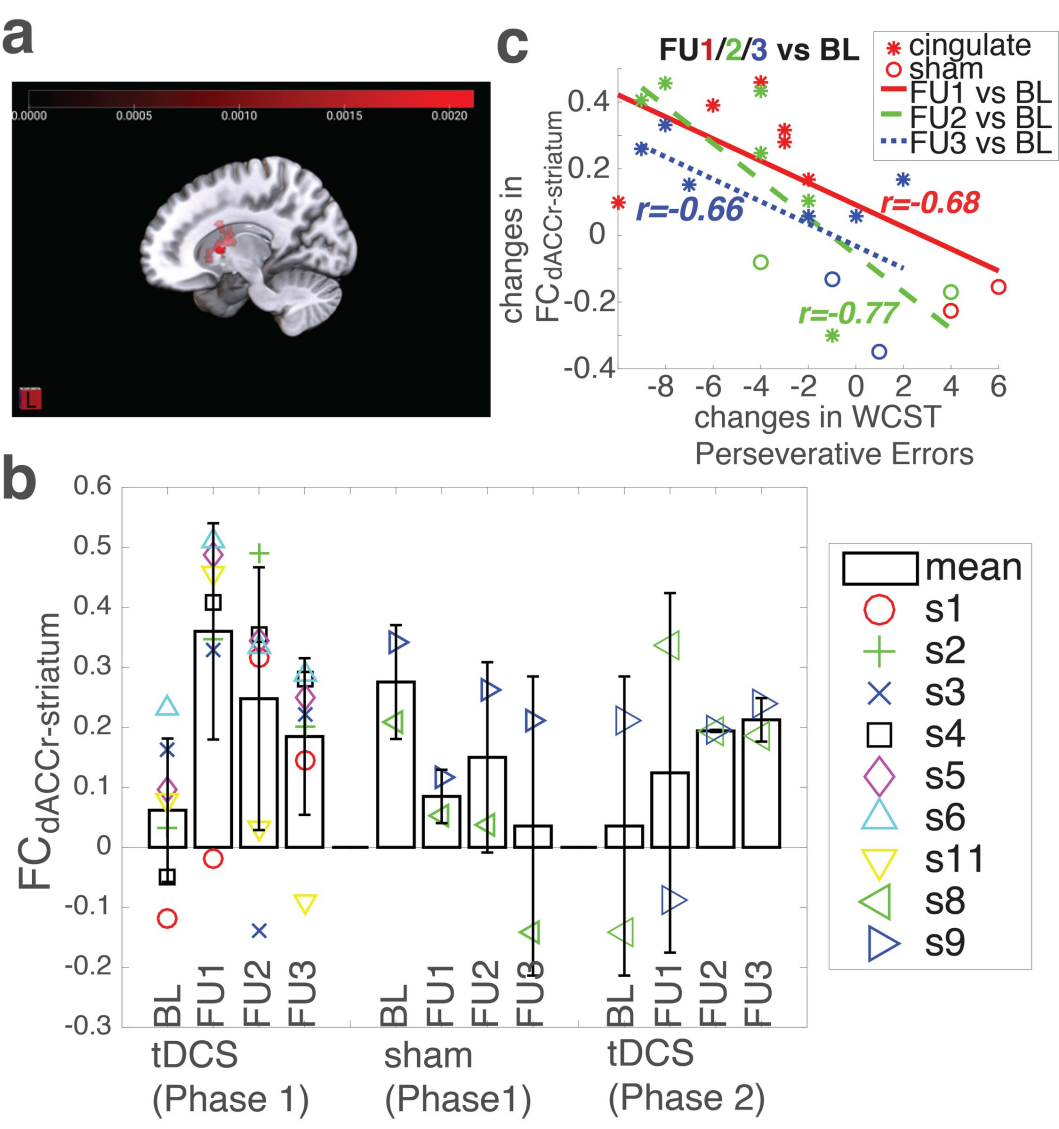
Resting state Functional Connectivity (FC). **(a)** Seed-to-voxel FC analysis was limited to participants who received active cingulate tDCS treatment during Phase 1. The bilateral dACC as the seed region revealed increased FC between the dACC and a cluster in the right dorsal striatum using the contrast of FU1 versus BL (MNI: [18 2 12], p < .05, FDR cluster-level correction). **(b)** The FCs between the bilateral dACC and the right dorsal striatum cluster (FC_dACC-rStriatum_) from all participants (markers represent individual participants in the same order as in Fig 2). Note there were no WCST data from subject s11, no MRI data from s7 due to MRI contraindications, and no FC data from s10 due to excessive head movement (S1 Table). Error bars represent standard deviation. **(c)** The correlations between changes in FC_dACC-rStriatum_ and changes in WCST Perseverative Errors during Phase 1 of the study. Red, FU1 vs BL; Green, FU2 vs BL; Blue, FU3 vs BL; *, participants who received active cingulate tDCS during Phase 1; o, participants who received sham tDCS during Phase 1.

Using the bilateral dACC as the seed region, seed-to-voxel analysis on 7 participants who received cingulate tDCS during Phase 1 with the contrast of BL vs FU1 revealed a cluster at the right dorsal striatum (MNI: [18 2 12], 1936 mm^3^) (Fig 4A). However, there were no significant results for the comparisons of BL vs FU2 or BL vs FU3. In addition, there were no significant results with the bilateral PCC as the seed region for any of the three comparisons.

Next, we extracted the FCs between the dACC and the right dorsal striatum cluster (FC_dACC-rStriatum_) from all participants and all visits (BL, FU1, FU2, FU3), including Phase 2 (if applicable) (Fig 4B). The potential associations between changes in WCST Perseverative Errors and changes in FC_dACC-rStriatum_ (FU1 vs BL, FU2 vs BL, and FU3 vs BL from Phase 1) were also examined. At all three follow-up visits (FU1, FU2, and FU3), the changes (compared to the baseline visit) in FC_dACC-rStriatum_ tended to correlate with the changes in WCST Perseverative Errors (Fig 4C).

## Discussion

In this randomized, “double-blind”, placebo-controlled, partial crossover cingulate tDCS pilot study, we examined the safety and potential efficacy of anodal tDCS neuromodulations over the dorsal ACC (dACC) and the PCC in adults with chronic HIV-disease.

The primary goal of this pilot study was to investigate the safety and tolerability of cingulate tDCS in PWH. Compared to other invasive (i.e., deep brain stimulation) and non-invasive (i.e., rTMS) brain stimulation techniques, tDCS is appreciated for its safety [44, 58, 59] and tolerability [59, 60]. In this pilot study, only one participant reported a burning sensation at one electrode location (CPz) after the first active tDCS session and the burning sensation went away after the anodal electrode was moved from CPz to Pz. In addition, self-reported discomfort ratings using the Wong-Baker FACES Pain Scale were comparable between participants who received sham tDCS and participants who received active cingulate tDCS (both during Phase 1), and between sham tDCS and active cingulate tDCS in participants who received both (sham tDCS during Phase 1 and active tDCS during Phase 2) (Table 1). Taken together, these data suggest that the current cingulate tDCS setup (1.5 mA anodal tDCS over dACC and PCC) is safe and can be well-tolerated by adults with chronic HIV-disease.

The secondary goal of this pilot study was to examine the potential effects of cingulate tDCS on improving cognitive function, with a focus on executive function, which is one of the most commonly affected cognitive domains in PWH in the cART era [25]. Of the four test scores from three standard neuropsychology tests that have been widely used to assess executive function (Fig 2), we found a significant interaction between treatment assignments (sham versus active cingulate tDCS) and study visits (BL vs FU1, and BL vs FU2) on WCST Perseverative Errors performance, suggesting that active cingulate tDCS may have led to a decrease in WCST Perseverative Errors, indicating an improvement in cognitive flexibility, an important component of executive functions. This notion is further supported by a similar but slightly weaker effect with the TMT-B / TMT-A ratio score–another measure of cognitive flexibility and executive control [50, 61]. Brain imaging [62] and brain lesions studies [63, 64] have provided converging evidence supporting an important role of dACC (in addition to DLPFC, especially the right DLPFC) in performing WCST, especially with regards to Perseverative Errors [63, 64], suggesting that the decrease in WCST Perseverative Errors might reflect an improvement in dACC neuronal function after cingulate tDCS. This hypothesis is supported by findings from previous magnetic resonance spectroscopy (MRS) studies. First, NAA is located exclusively in neurons and their processes; a decrease in NAA is believed to reflect either permanent neuronal loss or reversible neuronal/axonal dysfunction [65], suggesting that NAA concentrations might serve as a marker of neuronal/synaptodendritic injury in neurological diseases, including HAND [3]. Second, a previous study found that five consecutive sessions of anodal tDCS (2 mA) over dACC in adults with spinal cord injury led to a significant decrease in neuropathic pain, which negatively correlated with a significant increase in the concentrations of NAA (and glutamate/glutamine (Glx)) in the dACC–suggesting that anodal tDCS over the dACC might be effective in improving dACC neuronal function (i.e., an increase in dACC NAA concentrations) [66]. Third, in another study using high frequency (15 Hz) rTMS in adults with drug-resistant major depressive disorders [31], twenty sessions of rTMS over the left DLPFC led to a decrease in WCST Perseverative Errors and an increase in NAA concentrations in the dACC, and crucially, the changes in the two measures strongly correlated with each other, suggesting that a decrease in WCST Perseverative Errors might be attributed to an increase in dACC NAA, at least partially. Taken together, these findings support the notion that cingulate tDCS with the current setup (Fig 1C) may be effective in improving dACC neuronal function (i.e., an increase in dACC NAA), which in turn may lead to an improvement in cognitive flexibility–as supported by reduced WCST Perseverative Errors and a decrease in the ratio score of TMT-B / TMT-A. This notion can be directly tested in a future MRS study using a similar tDCS design.

In addition to directly modulating neuronal function within the stimulated brain region(s) (i.e., dACC), tDCS may impact/improve the functional brain networks associated with the stimulated brain region(s) [67, 68]. Indeed, a seed-to-voxel analysis revealed an increase in FC between the dACC and the right dorsal striatum (FC_dACC-rStriatum_) after cingulate tDCS (Fig 4A). This finding—while preliminary due to the small sample size (n = 7)—is interesting. As part of the corticostriatal circuitry, the extensive projections from the dACC to the dorsal striatum are well-established [69] and supported by data from diffusion tensor imaging [70] and resting state functional connectivity studies [71, 72]. Furthermore, a previous study found that the resting state FC between the dACC and the dorsal caudate negatively correlated with WCST Perseverative Errors in healthy adults [73], suggesting that effective communication between the dACC and the dorsal striatum is important to efficiently adjust response strategy (i.e., after receiving a negative feedback), probably by modulating neuronal activity in the dorsal striatum [74]. Therefore, the cingulate tDCS-induced increase in FC_dACC-rStriatum_ in this study might reflect an improved communication between the dACC and the dorsal striatum, which in turn contributed to the reduced WCST Perseverative Errors (Fig 4C), providing further evidence suggesting that, even though the location of stimulation with tDCS cannot be finely controlled and is never focal (Fig 1B), the current tDCS setup was effective in modulating/improving dACC function and may have the potential to improve brain function in PWH.

This study has several limitations. First, as a pilot study with a small sample size, this study lacks the power to determine the efficacy of cingulate tDCS; therefore, the preliminary findings with resting state FC as well as WCST and Trail Making Tests scores—while they are encouraging—should be taken with caution as a less stringent statistical approach was used (i.e., there was no correction for multiple comparisons for neuropsychological data analyses), which could lead to an increase in false positives. Nevertheless, the effect sizes in Fig 3 could be used to design a larger study that is powered to test for efficacy of this treatment approach. Second, other factors might also contribute to the observed improvement in neuropsychological test scores (i.e., reduced WCST Perseverative Errors as well as a decrease in TMT-B / TMT-A ratio), including a practice effect due to repeated administration of these neuropsychological tests, and/or the targeted cognitive training after each tDCS session. Third, due to the budget constraint, we used a partial crossover study design instead of a full crossover design, and the Phase 2 of the study was an open-label study that might have biased the results, though we did not include Phase 2 data in statistical analysis. Fourth, the demographics of the study sample might make it difficult to generalize the findings to the HIV+ community at large, i.e., all participants were African Americans with chronic HIV-disease (disease duration ranging from 16–36 years) and the vast majority of them were males (9 out of 11) and older than 54 (10 out of 11). However, it is of a significant interest to investigate whether the current tDCS setup may benefit HIV-infected youth, who are impaired in executive domains, “particularly in cognitive flexibility and inhibition” [75]. Future studies with a larger and more balanced (i.e., age, race, sex/gender, disease duration, etc.) sample, along with a double-blind and full crossover study design, are necessary to adequately assess the safety and efficacy of anodal tDCS over dACC and PCC on brain structure and function in PWH.

In summary, for adults with chronic HIV disease, anodal tDCS neuromodulation over the dACC and the PCC is safe and can be well-tolerated, and may have the potential to improve brain and cognitive function.

## Supporting information

S1 Table Demographics and treatment assignments of each individual participant. (DOCX)

S2 Table Neuropsychological data. (DOCX)
