# Correction: Cingulate transcranial direct current stimulation in adults with HIV

**DOI:** 10.1371/journal.pone.0305368

**Published:** 2024-06-06

**Authors:** Xiong Jiang, Sophia Dahmani, Margarita Bronshteyn, Fan Nils Yang, John Paul Ryan, R. Craig Gallagher, Srikanth R. Damera, Princy N. Kumar, David J. Moore, Ronald J. Ellis, Peter E. Turkeltaub

There are errors in the Author Contributions. The correct contributions are:

**Conceptualization:** Xiong Jiang.

**Data curation:** Margarita Bronshteyn, John Paul Ryan.

**Formal analysis:** Sophia Dahmani, Margarita Bronshteyn, Fan Nils Yang, John Paul Ryan, R. Craig Gallagher, Jr., Srikanth R. Damera.

**Funding acquisition:** Xiong Jiang, Princy N. Kumar, Peter E. Turkeltaub.

**Investigation:** Xiong Jiang, Margarita Bronshteyn, John Paul Ryan, Princy N. Kumar, David J. Moore, Ronald J. Ellis.

**Methodology:** Xiong Jiang, Peter E. Turkeltaub.

**Project administration:** Xiong Jiang, Margarita Bronshteyn, John Paul Ryan.

**Resources:** Xiong Jiang, Peter E. Turkeltaub.

**Supervision:** Xiong Jiang, Peter E. Turkeltaub.

**Visualization:** Xiong Jiang, Sophia Dahmani, Fan Nils Yang, R. Craig Gallagher, Jr.

**Writing–original draft:** Xiong Jiang, Sophia Dahmani.

**Writing–review & editing:** Xiong Jiang, Sophia Dahmani, R. Craig Gallagher, Jr., David J. Moore, Ronald J. Ellis, Peter E. Turkeltaub.
