# Supplementary material for: Cingulate transcranial direct current stimulation in adults with HIV
Source: PLoS One. 2022 Jun 3;17(6):e0269491. doi: 10.1371/journal.pone.0269491 (PMC9165807; doi:10.1371/journal.pone.0269491)
Supplement: S1 Table — (DOCX) [file pone.0269491.s001.docx]

**S1 Table**. **Demographics and treatment assignments of each individual participant.** This study used a partial crossover design and had two study phases: the first study phase (Phase 1) was double-blinded and the second study phase (Phase 2) was open-label. Among the eleven participants who finished the study, seven of them received active cingulate tDCS during Phase 1 and did not participate in Phase 2, whereas the other four received sham tDCS during Phase 1 then active cingulate tDCS during Phase 2.

| **^1^ID** | **Age** | **Sex** | **^2^Edu** | **^3^HAND** | **Disease Duration (years)** | **Viral load**  **(copies/ mL)** | **CD4**  **(cells/uL)** | **CD4 Nadir**  **(cells/uL)** | **^4^Phase 1 (tDCS/**  **sham)** | **^5^Phase 1 (pain scale)** | **^6^Phase 2 (tDCS/**  **sham)** | **Phase 2 (pain scale)** | **^7^NP** | **MRI** | **Resting State fMRI** |
| --- | --- | --- | --- | --- | --- | --- | --- | --- | --- | --- | --- | --- | --- | --- | --- |
| ^9^s1 | 54 | M | 14 | ^8^ANI | 34 | U | 672 | 200 | tDCS | N/A | - | - | Yes | Yes | Yes |
| s2 | 59 | M | 12 | ^10^NCN | 27 | U | 250 | 40 | tDCS | N/A | - | - | Yes | Yes | Yes |
| s3 | 57 | M | 13 | NCN | 31 | U | 689 | 500 | tDCS | N/A | - | - | Yes | Yes | Yes |
| s4 | 47 | F | 11 | NCN | 29 | U | 965 | 300 | tDCS | 0 (0) | - | - | Yes | Yes | Yes |
| s5 | 55 | M | 12 | NCN | 22 | U | 575 | 150 | tDCS | 0 (0) | - | - | Yes | Yes | Yes |
| s6 | 58 | M | 17 | NCN | 31 | U | 1094 | 497 | tDCS | 0.20 (0.42) | - | - | Yes | Yes | Yes |
| ^11^s11 | 69 | M | 16 | ^12^N/A | N/A | N/A | N/A | N/A | tDCS | 0 (0) | - | - | No | Yes | Yes |
| ^13^s7 | 62 | F | 16 | NCN | 25 | U | 605 | 131 | sham | N/A | tDCS | 0.60 (0.97) | Yes | No | No |
| s8 | 57 | M | 14 | NCN | 30 | U | 1360 | 100 | sham | 2.0 (0) | ^15^tDCS | 2.0 (0) | Yes | Yes | Yes |
| s9 | 55 | M | 14 | NCN | 28 | 6353 | 209 | 8 | sham | 0 (0) | ^15^tDCS | 0 (0) | Yes | Yes | Yes |
| ^14^s10 | 62 | M | 12 | NCN | 36 | 20 | 1181 | 18 | sham | 0 (0) | ^15^tDCS | 0 (0) | Yes | Yes | No |

^1^ID: the subject ID was in the same order as in Figure 2 and Figure 3 in the main article;

^2^Edu: education (years of formal education);

^3^HAND: HIV-associated neurocognitive disorders;

^4^Phase 1: the first phase of the study (double-blinded), in which participants were randomly assigned to receive active cingulate tDCS or sham tDCS treatment and the treatment assignments were unknown to the participants and the technicians who administered the tDCS;

^5^Phase 1 (pain scale): pain scale during both Phase 1 and Phase 2 was obtained through self-report using the Wong-Baker FACES visual analog pain scale, and was reported as mean (STD) of all ten tDCS sessions from each study phase. The pain scale data was missing from four participants (s1, s2, s3, and s7) for Phase 1;

^6^Phase 2: the second phase of the study (open-label), which was offered to participants who received sham tDCS during Phase 1;

^7^NP: the neuropsychology battery that was used for HAND diagnosis using the Frascati criteria;

^8^ANI: asymptomatic neurocognitive impairment;

^9^this participant (s1) was determined cognitively normal at FU2, but again met ANI criteria at FU1 and FU3;

^10^NCN: neurocognitively normal;

^11^s11: this subject did not have data from the neuropsychology battery for HAND diagnosis;

^12^N/A: Not applicable;

^13^s7: this subject did not participate in MRI portion of the project due to MRI contraindications;

^14^s10: this subject had excessive head movements during the resting state fMRI scan and was excluded from the resting state functional connectivity data analysis;

^15^tDCS: due to inclement weather (s8), illness (s9), or family emergency (s10), the three participants (s8, s9, and s10) received only 9 active tDCS sessions during Phase 2 after received 10 sham tDCS sessions during Phase 1. All other participants received 10 sham or active tDCS sessions during Phase 1 or Phase 2.
