# Supplementary material for: Cingulate transcranial direct current stimulation in adults with HIV
Source: PLoS One. 2022 Jun 3;17(6):e0269491. doi: 10.1371/journal.pone.0269491 (PMC9165807; doi:10.1371/journal.pone.0269491)
Supplement: S2 Table — (DOCX) [file pone.0269491.s002.docx]

**S2 Table. Neuropsychological data.** The data from the neuropsychology battery that was used for HAND diagnosis (median (IQR)).

| **NP Measure** | **Group** | **n** | **Baseline (BL)** | **Follow-Up 1 (FU1)** | **Follow-Up 2 (FU2)** | **Follow-Up 3 (FU3)** |
| --- | --- | --- | --- | --- | --- | --- |
|  |  |  | **Median (IQR)** | **Median (IQR)** | **Median (IQR)** | **Median (IQR)** |
| **WCST Perseverative Errors** | 1st: tDCS | 6 | 18.00 (3.50) | 13.00 (6.75) | 14.50 (1.75) | 14.00 (8.50) |
|  | 1st: Sham | 4 | 10.50 (6.00) | 12.00 (9.50) | 15.00 (6.00) | 15.50 (13.75) |
|  | 2nd: tDCS | 4 | 15.5 (13.75) | 12.00 (8.75) | 12.00 (9.25) | 10.50 (7.00) |
| **WCST Nonperseverative Errors** | 1st: tDCS | 6 | 14.50 (8.00) | 12.50 (5.75) | 11.50 (2.50) | 13.50 (7.50) |
|  | 1st: Sham | 4 | 9.50 (6.00) | 13.50 (8.50) | 9.00 (8.50) | 10.50 (6.75) |
|  | 2nd: tDCS | 4 | 10.50 (6.75) | 7.00 (5.50) | 7.50 (4.75) | 8.00 (10.25) |
| **STROOP Color-Words Total** | 1st: tDCS | 6 | 30.50 (18.75) | 30.00 (3.00) | 32.50 (17.75) | 34.00 (10.50) |
|  | 1st: Sham | 4 | 37.00 (10.75) | 37.50 (17.25) | 42.00 (18.50) | 38.50 (13.00) |
|  | 2nd: tDCS | 4 | 38.5 (13.00) | 28.50 (10.75) | 40.00 (13.00) | 39.00 (19.50) |
| **Trail Making Test - Part A (sec)** | 1st: tDCS | 6 | 28.00 (7.50) | 26.00 (14.50) | 28.00 (7.25) | 27.00 (10.75) |
|  | 1st: Sham | 4 | 42.00 (21.00) | 30.00 (7.50) | 22.50 (9.25) | 24.00 (15.00) |
|  | 2nd: tDCS | 4 | 24.00 (15.00) | 27.50 (8.75) | 28 (15.75) | 24.00 (13.00) |
| **Trail Making Test - Part B (sec)** | 1st: tDCS | 6 | 130.00 (92.00) | 88.50 (32.50) | 98.00 (29.00) | 75.00 (36.75) |
|  | 1st: Sham | 4 | 96.00 (47.75) | 91.00 (34.75) | 73.00 (35.25) | 81.50 (23.00) |
|  | 2nd: tDCS | 4 | 81.50 (23.00) | 82.50 (49.75) | 72.00 (35.00) | 86.50 (27.00) |
